# tsRNA: A Promising Biomarker in Breast Cancer

**DOI:** 10.7150/jca.93531

**Published:** 2024-03-11

**Authors:** Ting Huang, Yuexin Zhao, Guoqin Jiang, Zhixue Yang

**Affiliations:** 1 Department of Thyroid and Breast Surgery, The Second Affiliated Hospital of Soochow University, Suzhou, 215004, China.; 2 Department of Clinical Laboratory, The Second Affiliated Hospital of Soochow University, Suzhou, 215004, China.

**Keywords:** Breast Cancer, tRF, tiRNA, tsRNA, Biomarker, Liquid Biopsy

## Abstract

tRNA-derived small RNAs (tsRNAs) are a novel class of non-coding small RNAs, generated from specific cleavage sites of tRNA or pre-tRNA. tsRNAs can directly participate in RNA silencing, transcription, translation, and other processes. Their dysregulation is closely related to the occurrence and development of various cancers. Breast cancer is one of the most common and fastest-growing malignant tumors in humans. tsRNAs have been found to be dysregulated in breast cancer, serving as a new target for exploring the pathogenesis of breast cancer. They are also considered new tumor markers, providing a basis for diagnosis and treatment. This article reviews the generation, classification, mechanism of action, function of tsRNAs, and their biological effects and related mechanisms in breast cancer, in the hope of providing a new direction for the diagnosis and treatment of breast cancer.

## Introduction

tRNA derived small RNAs (tsRNAs) are a class of non-coding small RNAs, including tRFs (tRNA derived fragments) and tiRNAs (tRNA-derived stress-induced RNA)^1^. With the development of research technology, it has been discovered to be not random products of tRNA degradation but small RNAs that are cleaved according to specific sequences and are associated with various diseases^2^-^4^. Depending on the different cleavage sites of tRNAs, tRFs can be divided into tRF-1, tRF-2, tRF-3, tRF-5 and i-tRF, and tiRNA can be classified into 5'-tiRNA and 3'-tiRNA^2^, ^5^. Extensive research has confirmed that tsRNA can act as functional molecules, thereby affecting the progression of diseases^6^-^9^.

Breast cancer (BC) is a major threaten to women's health and one of the most prevalent malignant tumors worldwide^10^-^12^. The International Agency for Research on Cancer (IARC) released the GLOBOCAN 2020 data from 185 countries, which reported 2.26 million incident cases of breast cancer (11.7%), surpassing lung cancer as the most common cancer for the first time, and 680,000 mortality cases (6.9%)^13^. Therefore, investigating the pathogenesis of breast cancer and identifying potential biomarkers for its diagnosis and treatment are of great value and meaning.

This article reviews the recent advances in the biogenesis, classification, modification and biological functions of tsRNAs. Specifically, we focus on the effects and underlying mechanisms of tsRNAs on breast cancer cell proliferation, metastasis, apoptosis and other biological processes, aiming to explore their potential role in molecular diagnosis and targeted therapy for breast cancer.

## Biogenesis, classification, and detection methods of tsRNA

Pre-tRNAs are transcribed by RNA polymerase III, with a leader sequence and a tail sequence at their 5' and 3' ends. The leader sequence is subsequently removed by the endonuclease RNase P, which is konwn as a ribonucleoprotein, while the tail sequence is removed by RNase Z, through endo-nucleolytic cleavage exactly at the first unpaired base at the 3'-end of the tRNA^14^. Subsequently, the enzyme tRNA nucleotidyl transferase add a non-templated single “CCA” sequence to the 3'-ends of the trailer-free tRNAs^15^. Mature tRNAs have a length of 75-93nt and are conserved with four loops: the D-loop, anticodon loop, TψC loop and variable loop. With the progress of high-throughput sequencing technology, it has been found that tRNA or its precursor (pre-tRNA) can be cleaved by several enzymes at specific sites to generate tsRNAs. These non-coding small RNAs are widely distributed in prokaryotic and eukaryotic transcriptomes^16^-^18^. tsRNAs can be divided into different types, including tRFs and tiRNAs, depending on the cleavage site^19^, ^20^.

### tRFs

One group of small RNAs is 14-30 nucleotides in length and localized to the ends of mature or primary tRNA transcripts, are known as tRFs. tRFs can be mainly classified into five subclasses: tRF-1, tRF-2, tRF-3, tRF-5, and i-tRF. tRF-1 is a small fragment generated by the cleavage of the 3' end of the pre-tRNA by RNase Z or ELAC2. The pre-tRNA has a 3' tail sequence containing a poly-U sequence, and its 3' end matches the termination signal of RNA pol III^21^. tRF-2 is enzymatically cleaved from the anticodon arm of tRNA Tyr, tRNA Gly, tRNA Asp, or tRNA Glu. It contains stem sequence and anticodon loop, but lacks the 5' and 3' ends of tRNA^22^, ^23^. tRF-3 is a fragment of 18-22 nucleotides, containing the 3' terminal part of CCA sequence. It is produced by the cleavage of ANG (Angiogenin), Dicer or members of the ribonuclease family in the T-loop of mature tRNA. It can be divided into two subclasses: tRF-3a (-18nt) and tRF-3b (-22nt)^19^, ^24^. tRF-5 is a derivative fragment of less than 30 nucleotides, which Adenine is enriched at the 3' end. It is cleaved by Dicer at different cleavage sites in the D loop or the stem region between the D loop and the anticodon loop in tRNA. tRF-5 can be divided into three subtypes: tRF-5a (14-16nt), tRF-5b (22-24nt) and tRF-5c (28-30nt)^22^, ^23^. i-tRFs are internal segments located in mature tRNA, namely 36nt. They do not start at the first nucleotide of the 5' end of mature tRNA nor end at any base in the “CCA” sequence at the 3' end of mature tRNA^25^.

Most tRFs are generated by the cleavage of a single mature tRNA molecule, but some tRFs are seemed to be produced by the Dicer-mediated cleavage of misfolded pre-tRNA molecules, which can effectively degrade abnormal tRNAs^26^.

Dicer can cleave and generate various tRFs, but small RNA sequencing data from wild-type and Dicer mutants showed that Dicer mutation did not affect the expression of any tRFs in Drosophila, yeast, or mice^27^. Analysis of the 5' end of tRF-3 revealed that its cleavage site was between the A/U nucleotides of the tRNA single stranded loop, whereas tRF-5 lacked the specificity. However, they all have 5' - phosphate and 3 '- hydroxyl groups similar to miRNAs. Therefore, tRFs may be regarded as miRNAs, such as miR-1280, which was later confirmed to be a fragment of tRNA Leu^28^. Nevertheless, tRFs and miRNAs are distinct RNAs, because miRNAs depend on Dicer cleavage. This phenomenon suggested that there are unknown specific nucleases that need further investigation.

Interestingly, in the experiment of HeLa cell line, it is found that tRF-5 was mainly localized in the nucleus, while tRF-3 and tRF-1 were predominantly distributed in the cytoplasm. The underlying mechanism for this differential localization is still unclear. It may be related to the unknown enzymatic digestion mode or function of tRFs^27^.

### tiRNAs

tRNA halves are 31-40nt tRNA fragments that are produced by specific cleavage in the anticodon loop of mature tRNAs. They were first discovered in Tetrahymena thermophila, and later shown to be a conserved response to stress in eukaryotes^29^. Normally, they induced by stress (including heat shock, hypoxia, viral infection, etc.) Due to their stress-induced properties, they are also known as tiRNAs^23^, ^30^. The anticodon loop can be cleaved by several endonucleases, such as ANG, RNase L (vertebrates), Ryn 1 (yeast) and other that yet to be identified. Depending on the 5'- and 3'- sequences of the anticodon cleavage sites, tiRNAs can be classified into two subtypes: 5'-tiRNAs and 3'-tiRNAs. 5'- tiRNAs span from the 5'- end of the mature tRNA to the cleavage site on the anticodon loop, while 3'-tiRNAs span from their cleavage site on the anticodon loop to the 3'- end of the mature tRNA^31^. However, according to the detection, there seems to be no correlation between the tRNA gene copy number and the gene abundance of tRNA halves. CysGCA and AlaAGC have high copy numbers, but they are not preferentially processed into tsRNAs. In contrast, GlyCCC with a relatively low copy numbers produces a high abundance of tsRNAs^32^. The fragments of tiRNAs increase, when organisms are exposed to stress. In renal ischemia/reperfusion and cisplatin nephrotoxicity models, stress-induced tRNA cleavage and the productions of tiRNAs were detected in the damaged kidney^33^. 5'tiRNA-HIS-GTG is considered to be involved in the response process of tumor hypoxic microenvironment and is significantly up-regulated in colon cancer tissues^34^. When breast cancer cell lines are stimulated by hypoxia, tDR-0009 and tDR-7336 can show to be significantly upregulated^35^.

This process occurs by stress-induced specific ribonucleases, currently known as RNY in yeast and ANG in mammals^31^. Interestingly, overexpression of ANG in cells does not produce tiRNAs when cells are not stressed. However, under stress conditions, ANG overexpressing cells synthesize a larger amount of tiRNAs even under mild stress. This indicates that stress activates ANG-mediated tiRNA cleavage and inhibits protein translation^36^.

The cleavage on the anticodon loop mediated by ANG and others is known as canonical cleavage. At the same time, researchers have also discovered a non-canonical cleavage distant from the anticodon loop. In vivo, mitochondrial tRNAs tend to this cleavage, while ANG and Ribonuclease/angiogenin inhibitor 1 (RNH1) do not seem to regulate this cleavage. The cleavage site of this non canonical fragment appears to be in the TΨC‐loop and is partially regulated by M1A (N1-methyladenosine) demethylation of Alkbh1^37^.

Nonetheless, not all tiRNAs are produced by stress. Shozo's team discovered a sex hormone dependent tRNA half, called SHOT-RNAs. Aminoacylated mature tRNAs generate it via angiopoietin-mediated anticodon cleavage. It has two subtypes: 5'- SHOT-RNAs and 3'-SHOT-RNAs. The 5'-SHOT-RNAs contain half of the 5'tRNAs with phosphate at the 5' end and cyclic phosphate at the 3'end. The 3'-SHOT-RNAs contain the 3'tRNA half with a hydroxyl group at the 5' end and an amino acid at the 3'end. It is facilitated by sex hormones and their receptors. Although SHOT-RNAs and tiRNAs share the same biogenesis factors, they are different RNAs^38^.

### Methods used for tsRNA detection

With the application and development of high-throughput RNA sequencing, an increasing number of tRNA-derived fragments have been discovered^39^. High-throughput sequencing technology enables the detection of various types of RNA, such as miRNA, lncRNA, and tRNA etc.^40^. However, distinguishing tsRNA fragments from random degradation products of tRNA is a noteworthy consideration. Therefore, specific amplification primers can be designed for the specific detection of tRF and tiRNA through quantitative reverse transcription polymerase chain reaction (qRT-PCR)^41^. The expression of tsRNA can also be detected through Northern blotting^42^. However, qRT-PCR has always been unsuitable for community hospitals lacking professional equipment or laboratory settings. Consequently, Wu et al. developed a novel method using catalytic hairpin assembly (CHA) circuits and regularly interspaced short palindromic repeats (CRISPR) for the isothermal and target-initiated amplification detection of ts3011a RNA. In this detection method, the presence of the target tsRNA triggers the CHA circuit, converting the new DNA double strand into the secondary cutting activity of the CRISPR-associated protein (CRISPR-Cas) 12a, achieving cascade signal amplification. Moreover, they demonstrated for the first time that, compared with qRT-PCR, this method is less likely to produce aerosol contamination and has good consistency with the qRT-PCR detection results in serum samples, thereby providing a new, simple, isothermal, ultra-sensitive, target-initiated amplification method. However, they only conducted experiments in pancreatic cancer, and whether it can serve as a broad-spectrum detection method still needs further exploration^43^.

## Functions and mechanisms of tsRNAs

Increasing evidence shows that tsRNAs are not mere by-products of tRNA cleavage, and have diverse biological functions, such as modulate gene expression, translation and epigenetic modification by interacting with a variety of mRNAs or proteins^44^-^46^.

### Nascent RNA silencing

Recent investigations have elucidated that tsRNAs can downregulate target gene expression via a process known as nascent RNA silencing (NRS). Intronic macrogenomic analyses reveal a significant enrichment of tsRNA targets within early introns of genes. Dicer, an endoribonuclease, cleaves long RNA molecules into shorter fragments, thereby generating tsRNAs. These tsRNAs subsequently form a complex with Argonaute 2 (Ago2). This Ago2-containing silencing complex binds in a sequence-specific manner to the intronic regions of target genes, thereby inhibiting the transcriptional process of RNA polymerase and leading to a reduction in target gene expression. This process, termed nascent RNA silencing, represents a gene expression regulatory mechanism occurring within the cellular nucleus^47^.

### Transcriptional regulation

Research has revealed that the microRNA cluster miR-4521/3676 is associated with tRNA sequences, further substantiating their classification as tsRNAs. These entities have been found to coexist within complexes containing Piwi-like protein 2 (PIWIL2). However, it remains inconclusive whether they can be definitively characterized as PIWI-interacting RNAs (piRNAs)^48^. Then, it hase been hound that a specific 5'-tRF, derived from tRNA-Glu, known as td-piR (Glu), has the ability to bind with PIWIL4. This interaction facilitates the recruitment of SETDB1, SUV39H1, and heterochromatin protein 1β (HP1β) to the promoter region of CD1A. Consequently, this promotes the methylation of H3K9, ultimately leading to the suppression of CD1A transcription. This example illustrates the intricate mechanisms through which tsRNAs can regulate transcription^49^.

### Post-transcriptional regulation

Human PAR-Chip (Photoactivatable-Ribonucleoside-Enhanced Crosslinking and Immunoprecipitation) data show that tRF-5 and tRF-3 can bind to Ago proteins 1, 3, and 4. The Ago-tRF complex then binds to mRNA. Thus, tRF may play a role in transcriptional regulation like miRNAs^27^. A tRNA-derived fragment designated CU1276 was identified to have miRNA-like functional properties, such as DICER1-dependent biogenesis, association with Argonaute proteins, and repression of mRNA transcription in a sequence-specific manner. In lymphoma cells, it plays a role in inhibiting proliferation and modulating molecular responses to DNA damage^50^. Similarly, high-grade serous ovarian cancer (HGSOC) is one of the most common ovarian epithelial malignancies. Experiments show that tRF-003357 is significantly elevated in the serum of HGSOC patients and in the SK-OV-3 ovarian cancer cell model, compared to the control groups. It indicates that tRF-003357 can form a complex with Argonaute protein similar to miRNA, leading to the regulation of the expression of HMBOX1, which is a transcription factor down-regulated in HGSOC tissues and cell^51^.

Furthermore, tsRNAs can regulate post-transcriptional gene expression by directly binding to RNA-binding proteins (RBPs). It has been found that in mouse models, a specific 5'-tsRNA can interact with the RNA-binding protein Igf2bp1 (Insulin-like growth factor 2 mRNA-binding protein 1), affecting the transcriptional stability, which in turn affected the translation of the pluripotency-promoting factor c-Myc^32^.

Meanwhile, tsRNAs can exert post-transcriptional regulation through various mechanisms. For instance, in a rat model of obesity induced by a high-fat diet, a small non-coding RNA derived from glutamine tRNA, known as tRF GluTTC, has been identified. This tsRNA can bind to the 3'UTR of KLF family genes (a group of zinc-finger transcription factors), thereby affecting mRNA stability, inhibiting its translation, and ultimately promoting fat deposition^52^. There are many other similar examples. It has been reported that in colorectal cancer, tRF-20-M0NK5Y93 promotes cancer cell migration and invasion by regulating Claudin-1 expression during EMT (Epithelial-to-mesenchymal transition). It can bind to the complementary sequence of Claudin-1 mRNA, leading to mRNA degradation or translation inhibition, and thus reduces Claudin-1 protein level^53^. tRF-Gly-GCC in sperm can regulate the expression of transcripts driven by endogenous transcription factors, delay or inhibit the MERVL (Murine Endogenous Retrovirus-L) expression in 2-cell embryos, affecting the size or function of the placenta, leading to metabolic effects due to altered placentation^54^. Due to the complexity of modifications and functions of tRNA-derived fragments, it affects tumor progression through diverse mechanisms at the same time. AS-tDR-007333, enhances the proliferation and invasion of non-small cell lung cancer (NSCLC) cells by activating two pathways. It can activate the interaction between HSPB1 (heat shock protein family B member 1) and MED29 (Mediator Complex Subunit 29) to promote the proliferation and invasion of NSCLC. At the same time, it can also up-regulate MED29 by activating ELK4-mediated transcriptional regulation to promote the proliferation and invasion of cancer cells^55^.

### Translational regulation

The regulation of translation by tsRNA can be either positive or negative. For instance, a 22nt-length LeuCAG3'-tsRNA can bind to ribosomal protein mRNAs such as RPS28 (Ribosomal protein 28) and RPS15, enhancing their translation and affecting the number of 40S ribosomal subunits. In mouse models, knockdown of LeuCAG3' tsRNA can induce massive apoptosis in HeLa cells and patient-derived orthotopic Hepatocellular carcinoma (HCC) xenografts^56^. 5'tRF-Gln-19 can interact with certain mammalian polysynthetase complexes, increase translation of ribosomes and poly(A)-binding proteins^57^.

Bellodi's group found that mini TOG (mTOG)-containing 5'-tRFs can be driven by PUS7 (Pseudouridine synthase 7)-mediated Ψ modification at U8 in embryonic stem cells to bind to PABPC1 (Polyadenylate-binding protein 1). The binding forms the mTOG-PABPC 1 complex, which can block the recruitment of PABPC 1 to eIF4F (Eukaryotic translation initiation factor 4F), thereby inhibiting protein translation^58^.

Aberrant methylation of tRNAs, caused by the lack of NSun2-mediated methylation at cytosine-5, impairs the survival and development of neurons in regions such as the cortex, hippocampus and striatum, leading to neuro-developmental disorders such as microcephaly, intellectual disability and behavioral defects. This aberrant methylation renders tRNAs more prone to cleavage by angiogenin (ANG) into 5'tiRNAs^59^. These tRNA fragments can inhibit global translation by displacing eukaryotic initiation factors eIF4G and eIF4A from mRNA and eIF4F from the detached m7G cap, thereby interfering with protein synthesis and reducing translation levels^60^.

Among various tsRNAs that can inhibit translation, 5'-tiRNAAla and 5'-tiRNAACys seem to be particularly effective translation inhibitors, possibly due to their multiple consecutive guanine residues (4-5). At low molar concentrations, 5'-tiRNAAla interferes with eIF4G function and its binding to the 5'-end of mRNA^44^. Further studies have demonstrated that translationally active 5'-tiRNA can form a G-quadruplex and then bind to the cold shock domain of YB-1, displacing the cap-binding complex eIF4F from mRNA and inhibiting translation initiation. Cell experiments demonstrated that 5'-tiRNA can partially replace eIF4E in the capped mRNA, and completely replace eIF4G/A. This inhibition of protein translation depends on the strength of eIF4G: RNA interaction. Strong interactions between the cap-binding complex and mRNA, such as UA6, prevent tiRNAs from inhibiting translation, while weak interactions, such as UA7, allow tiRNAs to inhibit translation^61^. However, it is confusing that among tiRNAs, only the 5'-tRNA halves can repress protein translation, while the 3'-tRNA halves cannot. This may be due to the extra V region and the hydroxyl group at the end of 3'-tiRNA^62^.

### Regulation of reverse transcription

Reverse transcription is the process of converting RNA into DNA, which is how some retroviruses replicate. The RNA genome of retroviruses contains a PBS (primer binding site), which can provide the starting point for the reverse transcription reaction^63^. In HTLV-1 (Human T-cell Leukemia Virus Type 1), many tRNAs-related fragments are expressed, among which the most abundant is tRF-3019 derived from the 3' end of tRNA-proline. Usually, HTLV-1 uses tRNA-Pro as a reverse transcription primer, but in some cases, tRNA-Pro is cleaved into tRF-3019, which can also bind to PBS. Because tRF-3019 is shorter than tRNA-Pro, it can pair more tightly with PBS, increasing the efficiency and stability of the reverse transcription reaction. In addition, tRF-3019 can also inhibit the competition of other tRNAs for PBS, increasing its advantage as a reverse transcription primer^64^.

Correspondingly, tRFs are not simply fragments that promote reverse transcription. Their regulatory effects on reverse transcription can be positive or negative. LTR (Long terminal repeat) retrotransposons are transposable elements with long terminal repeats, which are similar to retroviruses in structure and replication process^65^.

In mouse stem cells, abundant tRFs are abundant can target and inhibit the reverse transcription and transposition of LTR retrotransposons or endogenous retroviruses (ERVs). The 17-19 nt tRF-3a can fully or partially complement the PBS of ERVs, interfere with the reverse transcription process of ERVs, and prevent cDNA synthesis. Besides, the 22 nt tRF-3b can also bind to AGO protein, guiding RISC complex to mRNA, resulting in its degradation or translation inhibition^66^.

### Orchestrating cell cycle and apoptosome dynamics

In addition to affecting RNA silencing and protein translation, tRFs also regulate apoptosis. One example is tRF-1001, which is derived from the 3'-end of the Ser-TGA tRNA precursor transcript. When tRF-1001 is depleted, the number of viable cells decreases, cell proliferation is impaired, and it leads to an accumulation of cells in G2 phase of the cell cycle^22^. Similarly, when researchers transfect NSCLC cells with a tRF-Leu-CAG inhibitor, they observe that the number of cells in the G0/G1 phase increases and the cell proliferation capacity decreases^67^. In vivo, tiRNA can bind to Cyt c (Cytochrome c) released from mitochondria during hyperosmotic stress. This binding leads to the formation of a Cyt c-tiRNAs complex mediated by ANG. The complex can inhibit the formation of apoptosomes and protect cells from apoptosis during stress^68^. It is demonstrated that the tsRNA-cyt c complex can inhibit the combination of tsRNA with Apaf-1 (Apoptotic protease activating factor 1). This inhibition can block the activation of caspase-9 and the formation of apoptotic bodies, thereby inhibiting apoptosis.

## Dysregulation of tsRNAs in BC

Thus, dysregulation of tsRNA quantities can be found in a variety of different diseases. For instance, tRNA-GlyGCC-5 and sRESE are significantly upregulated in esophageal carcinoma. A type of 5'-tiRNA with a length of about 30-35nt exists in large quantities in chronic viral hepatitis, while its abundance is reduced in cancer tissues. Moreover, serum hsa\u-tsr016141 can clearly distinguish GC patients from healthy blood donors or gastritis patients, and is positively correlated with the degree of lymph node metastasis and tumor grade, so can be used as a new biomarker for GC diagnosis and postoperative monitoring^67^, ^69^-^71^.

Here, we focus on dysregulation of tRFs in BC. At the cellular level, tRF expression levels increase significantly under hypoxic stress in breast cancer cells, as preciously described^72^. High-throughput sequencing of non-triple-negative breast cancer cell lines and patient serum revealed that tDR-7816 was significantly upregulated in both cell lines and patient serum, while tDR-5334 and tDR-4733 were both downregulated^73^.

Plasma samples from breast cancer patients and healthy individuals were analyzed by small RNA high-throughput sequencing and qRT-PCR. The analysis revealed that tRF-Arg-CCT-017, tRF-Gly-CCC-001 and tiRNA-Phe-GAA 003 were significantly increased, and their expression varied across different subtypes. Specifically, tRF-Arg-CCT-017 was significantly higher in HER-2 subtypes, while tRF-Gly-CCC-001 and tiRNA-Phe-GAA-003 showed differences between Luminal and triple-negative breast cancer (TNBC)^74^. In addition, Wang et al. found that tRF-Glu-CTC-003, tRF-Gly-CCC-007, tRF-Gly-CCC-008, tRF-Leu-CAA-003, tRF-Ser-TGA-001 and tRF- Ser-TGA-002 were down-regulated in the plasma of early breast cancer patients. Notably, HER-2 positive EBC (early breast cancer) patients with high expression of tRF-Glu-CTC-003 had worse DFS (disease-free survival) and OS (overall survival)^75^.

Involving of TNBC, high-throughput sequencing was performed on the TNBC cell line MDA-MB-231 CSCs and the non-TNBC cell line MCF-7 CSCs, in order to identify differences in tDR expression between TNBC and non-TNBC cell lines. The results showed that 72 candidate tDRs with high counts and differential expression between two group CSCs. Among the differentially expressed tDRs, tDR-000620 expression was correlated with age, nodal status and recurrence^76^. A novel tsRNA, tRF Lys-CTT-010, was discovered in TNBC and found to be critically upregulated in TNBC tissues and promoted the proliferation and migration of TNBC cells^77^.

## Biological functions of tsRNA in BC

Breast cancer is a common and malignant tumor that resulted from the abnormal proliferation and invasion of breast cells. Its pathogenesis is influenced by various genetic and environmental factors, as well as the regulation of endocrine, immune, metabolic and microenvironmental aspects, etc.^78^.

tsRNAs have been shown to play roles in BC tumorigenesis and progression through various mechanisms. Estrogen exposure is a major risk factor for breast cancer. It can induce breast cancer by metabolizing into genotoxic and mutagenic substances, and by stimulating tissue growth. Together, these processes lead to the initiation, promotion, and progression of carcinogenesis. Notably, 70-75% of breast cancers express estrogen receptor-alpha, which contributes to estrogen-dependent tumor growth^79^. In studies of ER+ breast cancer, researchers have discovered that sex hormones and their receptors regulate SHOT-RNA production by activating ANG to cleave aminoacylated mature tRNA, and the accumulation of the resulting SHOT-RNA contributes to breast cancer cell proliferation and tumorigenesis. However, the mechanisms underlying the enhancement of cancer cell proliferation require further investigation^38^. Meanwhile, tRF-19-W4PU732S has been reported to enhance the activity of breast cancer cells. It has been found to be upregulated in breast cancer tissues and cells. Subsequent cellular experiments revealed that in the estrogen receptor-positive MCF7 cell line, tRF-19-W4PU732S post-transcriptionally regulates the expression of RPL27A. It exerts a targeted inhibition on the synthesis of RPL27A protein by affecting the synthesis of RPL27A mRNA, thereby promoting the proliferation, migration, and invasion of breast cancer cells^80^.

It is noteworthy that transfer RNA-derived small RNAs (tsRNAs) have been extensively reported to influence the onset and progression of breast cancer at the post-transcriptional level.

It was found that the abundance of a derivative fragment, 5'-tRFCys, increased in breast cancer cell lines. Experimental validation in MDA-MB-231 and 4T1 cells confirmed that 5'-tRFCys could bind with Nucleolin, promoting its oligomerization, and assemble it with metabolic transcripts Mthfd1l and Pafah1b1 into a more stable ribonucleoprotein complex. This protects these transcripts from exonuclease degradation, thereby driving cancer progression at the post-transcriptional regulatory level^81^. Chen et al. identified a specific tRNA-derived fragment, 5'-tRF-GlyGCC, which is upregulated in human breast cancer. This fragment directly interacts with the fat mass and obesity-associated protein, thereby enhancing the activity of FTO demethylase. Consequently, this post-transcriptional modulation reduces the methylation of eIF4G1, impacting translation, inhibiting autophagy, and promoting the proliferation and metastasis of breast cancer^82^.

Indeed, tsRNAs are not solely promoters of cancer progression. Goodarzi et.al identified a class of tRFs derived from tRNA (Glu), tRNA (Asp), tRNA (Gly), and tRNA (Tyr). These tRFs can displace cancer transcripts' 3' untranslated regions (UTRs) by binding with the RNA-binding protein YBX1, thereby suppressing the stability of these transcripts through sequence-specific transcriptional silencing, which in turn inhibits the progression of breast cancer^72^. Similarly, the expression of 5'-tiRNAVal is significantly reduced in breast cancer tissues. Experimental findings reveal that it modulates the stability of FZD3 mRNA, thereby post-transcriptionally inhibiting the FZD3-mediated Wnt/β-Catenin signaling pathway. Its downregulation is positively correlated with the staging of breast cancer tumors and lymph node metastasis^83^. An analogous phenomenon is observed with tRF-17-79-MP-9-PP (tRF-17), a 5'-tRF fragment that is expressed at lower levels in breast cancer tissues and serum. Overexpression of tRF-17 can inhibit the invasion and migration of breast cancer cells by targeting and suppressing the THBS1 (Thrombospondin-1) mediated TGF-b1/Smad 3 signaling pathway^84^. Wang et al. identified a tRNAiMet-derived, piR_019752-like 31-nucleotide fragment, tRiMetF31, which is downregulated in breast cancer cell lines. This fragment significantly inhibits angiogenesis and cancer cell migration through the silencing of PFKFB3. This discovery provides a novel perspective on the molecular mechanisms of breast cancer progression and offers potential therapeutic targets^85^.

The Human Epidermal Growth Factor Receptor 2 (HER-2), a receptor tyrosine kinase erbB-2, typically participates in the proliferation and division of breast cells. Under certain aberrant conditions, the HER2 gene malfunctions, leading to an overproduction of itself. HER2-positive (HER2+) breast cancer is a form of invasive breast cancer that tends to grow more rapidly and is more likely to spread^86^. Studies have revealed a significant reduction in the levels of tRF 3E in the blood of patients with HER-2 positive breast cancer. Experimental evidence suggests that tRF 3E forms a complex with nucleolin (NCL) through specific binding. This complex competitively binds with p53 mRNA, thereby preventing NCL from inhibiting the translation of p53 mRNA, ultimately suppressing the growth of cancer cells^87^.

Cellular heterogeneity in breast disease depends on the primary developmental sequence of the common mammary gland. Breast cancer heterogeneity may result from neoplastic changes in myoepithelial or epithelial cells or stem cells that can differentiate into cancerous myoepithelial or epithelial cells^88^. The RUNX1 (RUNX family transcription factor 1) transcription factor is a tumor suppressor in the mammary epithelium, and ts-112 is confirmed to have carcinogenic potential. Experiments found that RUNX 1 can prevent hyperactive cell proliferation by inhibiting ts-112, thereby enhancing its role in maintaining mammary epithelium. Nonetheless, a more comprehensive understanding of the mechanisms necessitates additional research^89^.

tsRNAs not only influence the onset of cancer at the molecular biology level, but also exert regulatory effects at the metabolic level. For example, tRFLys-CTT-010 was significantly increased in human TNBC. It interacts with the Glucose 6-Phosphatase catalytic subunit (G6PC) to regulate glycogen consumption and cellular lactate production, resulting in enhanced cell proliferation of TNBC^77^.

## The value of tsRNA in the diagnosis and treatment of BC

### Mechanistic factors of tsRNAs to become potential biomarker

The main methods for breast cancer screening are imaging tests (such as mammography, ultrasound, MRI, etc.), while the diagnosis relies on tissue biopsy^90^-^92^. The molecular markers of breast cancer involve TNM staging, immunohistochemistry results, gene testing results, tumor marker detection, etc., which are closely related to the diagnosis and prognosis of breast cancer^10^, ^93^-^95^. However, most of these methods require tissue samples, which means invasive in-hospital tissue biopsies. In addition, the specificity of routine tumor markers for women is not high. Therefore, there is an urgent need for a molecular marker with high sensitivity and specificity in clinical practice, which can reduce the trauma of patients and improve their quality of life.

In the circulating bloodstream, tRNA-derived fragments achieve protection from RNase degradation predominantly through encapsulation or association with extracellular RNA carriers.

Exosomes are membrane-bound nanovesicles released by both healthy and pathological cells^96^, containing a variety of biomolecules such as nucleic acids, proteins, and lipids, thereby promoting intercellular communication. Previous studies have demonstrated that exosomes contain numerous small non-coding RNAs, including miRNAs, lncRNAs, and circRNAs, which interact with recipient cells via exosomes, regulating the onset and progression of diseases. Recent studies have demonstrated that tsRNAs are also abundantly present in microvesicles or exosomes^97^. It has been discovered that during the activation process of T cells, T cells release specific tRFs into exosomes via multivesicular bodies, thereby removing the inhibitory effect of tRFs on immune activation^98^. Moreover, tsRNAs can be transferred from transfected cells to recipient cells via exosomes, and exert their functions through this specialized delivery system^99^. Zhu et.al have elucidated that the concentrations of four tsRNAs—tRNA-ValTAC-3, tRNA-GlyTCC-5, tRNA-ValAAC-5, and tRNA-GluCTC-5—manifest a pronounced augmentation within the plasma exosomes of individuals afflicted with hepatic carcinoma, heralding their prospective utility as innovative biomarkers for the diagnostic process of this malignancy^100^. Similarly, the expression levels of tRF-3022b, tRF-3030b, and tRF-5008b are markedly elevated in the plasma exosomes of colorectal cancer (CRC) patients, endowing them with potential clinical significance for the diagnosis and treatment of CRC^101^. The current absence of a standardized protocol for exosome isolation presents a significant challenge; devising a method to extract exosomes with high purity without compromising and degrading the concentration of non-coding small RNAs remains an area meriting rigorous investigation^102^.

However, most tRNA-derived fragments circulating in the bloodstream are not found inside EVs. Investigations have revealed that the majority of extracellular tRNA-derived fragments circulating in serum appear to be associated with extra-vesicular or ribonucleoproteins^103^. In the course of surveying small RNAs present in the serum of healthy adult humans, Joseph Dhahbi and his colleagues discerned a sequence peak within the range of 30-33 nucleotides, the majority of which (67%) mapped to tRNA. These fragments were subsequently identified as 5'-tiRNAs, and it was established that most are not associated with extracellular vesicles. Additionally, it was ascertained that these tRNA halves are transported in the bloodstream as complexes^104^. Such results have also been corroborated in other studies^105^-^107^. In the context of chronic lymphocytic leukemia, it has been discerned that TS-3676 and TS-4521 are significantly downregulated in the blood samples of tumor-afflicted patients. These tsRNAs are capable of interacting with Piwi-like protein 2 (PIWIL2)—a gene implicated in gene silencing and other regulatory functions—thereby assuming a pivotal role in the oncogenesis and progression of tumors^48^.

The aforementioned mechanism contributes to the abundant presence of tsRNA within the bloodstream, offering a promising outlook for its application as a minimally invasive blood biomarker^108^. In lung adenocarcinoma, it has been observed that the expression of tRF-16-L85J3KE, tRF-21-RK9P4P9L0 and tRF-16-PSQP4PE were significantly elevated. The combination of these three tsRNAs enhance the predictive accuracy for cancer prognosis, as evidenced by an increased AUC value of 0.92. Furthermore, patients exhibiting high expression levels of tRF-21-RK9P4P9L0 demonstrate a reduced overall survival duration^109^.

Notably, tsRNA also exhibits a rich concentration across various biological fluids, including urine, saliva, and bile, underscoring its potential as a universal biomarker. In patients with esophageal squamous cell carcinoma (ESCC), the salivary exosomes exhibit a specific enrichment of tRNA-GlyGCC-5 and sRESE. This dual biomarker signature distinguishes ESCC patients from the control group with high sensitivity (90.50%) and specificity (94.20%)^69^.

### Clinical applications of tsRNA in breast cancer

Therefore, tsRNA can serve as a promising biomarker for tumorigenesis, development, and clinical therapeutic target, on account of its noninvasive nature.

Huang et al. identified differentially expressed tRFs in normal and breast cancer cell lines, finding that tDR-7816 was critical upregulated, and its serum expression level was positively correlated with tumor size, lymph node metastasis, TNM stage and Ki-67 index. tDR-7816 expression could serve as a novel potential biomarker for the diagnosis of patients with early non-TNBC. The ROC analysis showed that tDR-7816 had a high diagnostic efficiency, with an area under the curve (AUC) of 0.892, a sensitivity of 0.867 and a specificity of 0.833^73^. Similarly, tRF-Gly-CCC-046, tRF-Tyr-GTA-010 and tRF-Pro-TGG-001 were down-regulated in breast cancer and early breast cancer. They had high diagnostic efficiency, with AUCs of 0.7223, 0.7809, 0.7085, and 0.7871 (combination), respectively, which can be used as potential biomarkers for the diagnosis and early diagnosis of breast cancer^110^.

tsRNAs have been confirmed to be associated with drug resistance in breast cancer and can serve as a biomarker for efficacy evaluation. Studies have shown that patients with high expression of tRF-30-JZOYJE22RR33 and tRF-27-ZDXPHO53KSN respond poorly to trastuzumab compared to patients with low expression of these molecules. tRF-30-JZOYJE22RR33 and tRF-27-ZDXPHO53KSN could be potential biomarkers (with ROC curves of 0.7147 and 0.8308, respectively) and intervention targets for clinical treatment of trastuzumab-resistant breast cancer^111^. Research by Sun et al. found that compared to tamoxifen-sensitive cells, the expression of tRF-16-K8J7K1B is upregulated in tamoxifen-resistant cells. High expression of tRF-16-K8J7K1B is associated with a shorter disease-free survival period in hormone receptor-positive (HR+) breast cancer. tRF-16-K8J7K1B in exosomes could potentially serve as a predictive biomarker and therapeutic target for overcoming tamoxifen resistance^112^. Researchers used high-throughput sequencing to compare the aberrant expression of tDRs in TNBC cell lines treated with hypoxia. They found a potential mechanism that upregulated tDR-0009 and tDR-7336 might promote doxorubicin resistance in TNBC by interfering with the maintenance of stem cell populations and cellular responses to interleukin (IL)-6^35^.

tsRNAs have also been shown to be associated with prognosis in breast cancer. Patients with low expression of tDR-000620 and lymph node metastasis have significantly shorter RFS (Recurrence Free Survival) than patients with high expression of tDR-000620 and negative lymph nodes. tDR-000620 can serve as an independent poor prognostic factor for recurrence-free survival in TNBC, and a new candidate biomarker for early detection of recurrence in TNBC patients^76^. Similarly, patients with high levels of tRF-Arg-CCT 017 or tiRNA-Phe-GAA-003 had poorer DFS and OS, thus can act as prognostic biomarkers^74^.

## Conclusion and perspectives

The development and application of high-throughput transcriptome sequencing has enabled the growing discovery of new tRFs. These classes of fragments, once considered as nonsense degradation products of tRNAs, has attracted renewed attention. Further research has revealed that these tRNA-derived fragments are cleaved with strict sequence specificity into tiRNAs and tRFs. They can further be classified based on the sequence part of the mature tRNA contained into 5'-tiRNA, 3'-tiRNA, tRF-1, tRF-2, tRF-3, tRF-5, and i-tRF.

tRFs are involved in key biological processes, such as nascent RNA silencing, transcriptional repression, post-transcriptional gene silencing, translational regulation, apoptosis suppression, etc. This article summarizes the research progress on tsRNA production, classification, biological function, and diseases, with a focus on their characteristics in breast cancer.

tsRNAs exhibit significant expression differences between cancer patients and healthy individuals, thus demonstrating excellent sensitivity and specificity. They can be released into body fluids via extracellular vesicles and can protect themselves from degradation by binding with RBPs or through extracellular vesicles, thereby ensuring their stable presence in body fluids. They can be detected through non-invasive or minimally invasive methods. tsRNA has been confirmed to have a large number of abnormal regulations, and participate in the occurrence, progression and drug resistance process of breast cancer. It affects the early treatment, recurrence and improvement of prognosis of breast cancer. Therefore, tsRNAs can serve as biomarkers for the diagnosis and treatment of breast cancer.

However, there are still some limitations in the research field of tsRNA. tsRNA may be affected by many factors, such as individual differences, diet, drugs, etc., thus need more clinical studies to exclude the influence of factors. Also, the biosynthesis mechanism and functional mechanism of tsRNA are not fully understood, thus need more experimental validation. High-throughput sequencing is the main method for detecting tsRNA, but it has a higher cost than traditional tumor markers (e.g., CEA and CA199) and imaging examinations (e.g., X-ray and B-ultrasound). Although tsRNA researches are advancing rapidly, current researches in breast cancer remain limited, and there is still a lack of clinical research verification with large sample size. Thus, the specific mechanism of action between tsRNAs and breast cancer remains to be further investigated.

In summary, this paper aims to summarize the current research on the production, classification, functional mechanism and role of tsRNA in breast cancer. We present tsRNA as a promising new class of non-coding small RNA, which has potential applications in the diagnosis and treatment of breast cancer. tsRNA can serve as a new candidate biomarker for liquid biopsy of breast cancer, as it shows large expression differences between cancer patients and normal people, and has high sensitivity and specificity. tsRNA can also act as a new molecular target for the treatment of breast cancer, as it is involved in the occurrence, progression and drug resistance of breast cancer, and affects the early treatment, recurrence and prognosis of breast cancer. This provides new targets and ideas for anti-tumor research.

## Funding

This study was supported by grants from Maternal and Child Health Research Project of Jiangsu Province (F202036). This study was also supported by Suzhou Health Talent Research Fund (GSWS2021024).

## Figures and Tables

**Figure 1:**
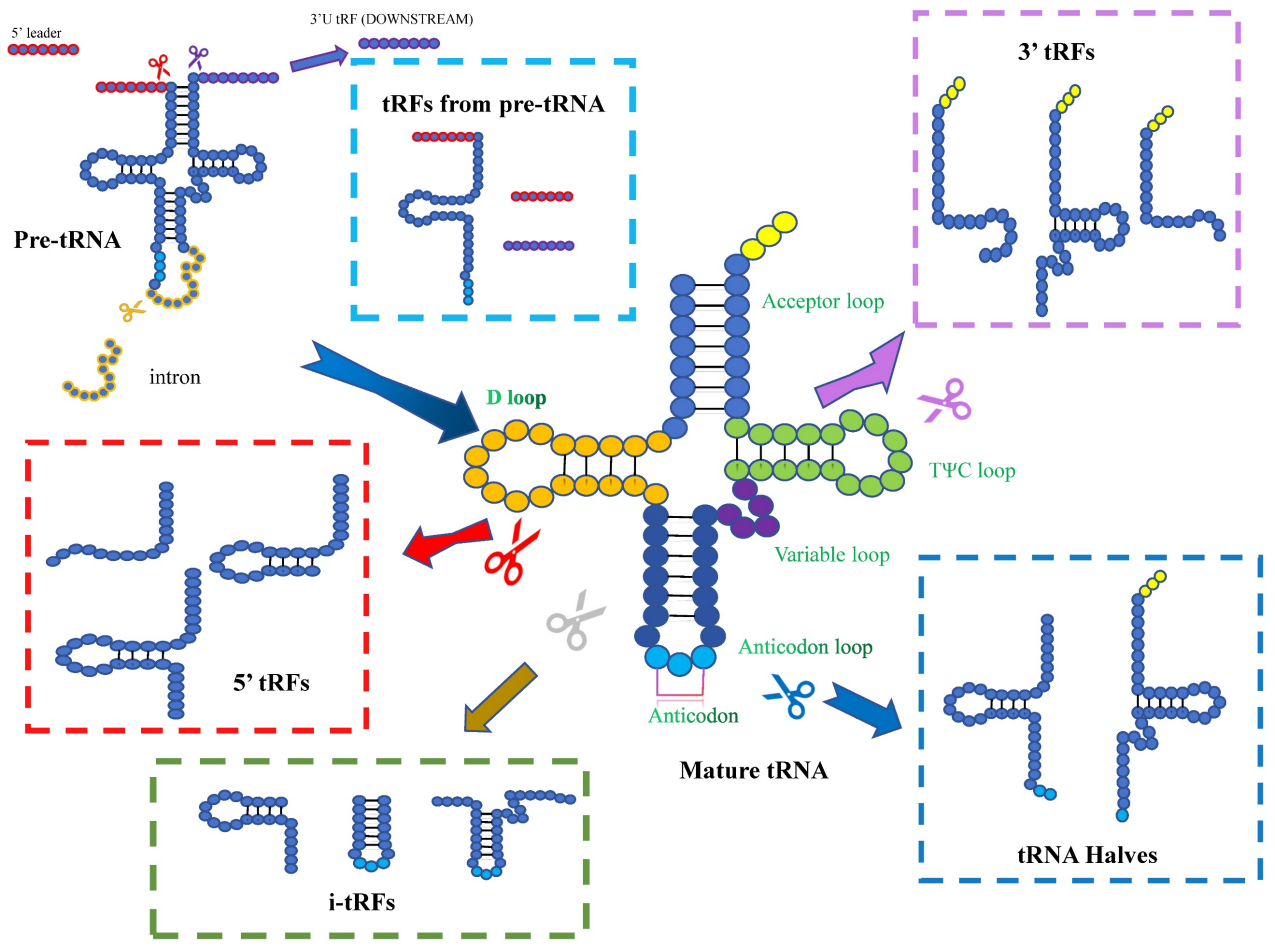
Biogenesis and classification of tsRNAs. Based on the cleavage site of mature tRNAs, tsRNAs can be classified into 5'-halves, 3'-halves, 5'-tRFs, 3'-tRFs, and i-tRFs. The cleavage of pre-tRNAs can also generates tsRNAs.

**Figure 2:**
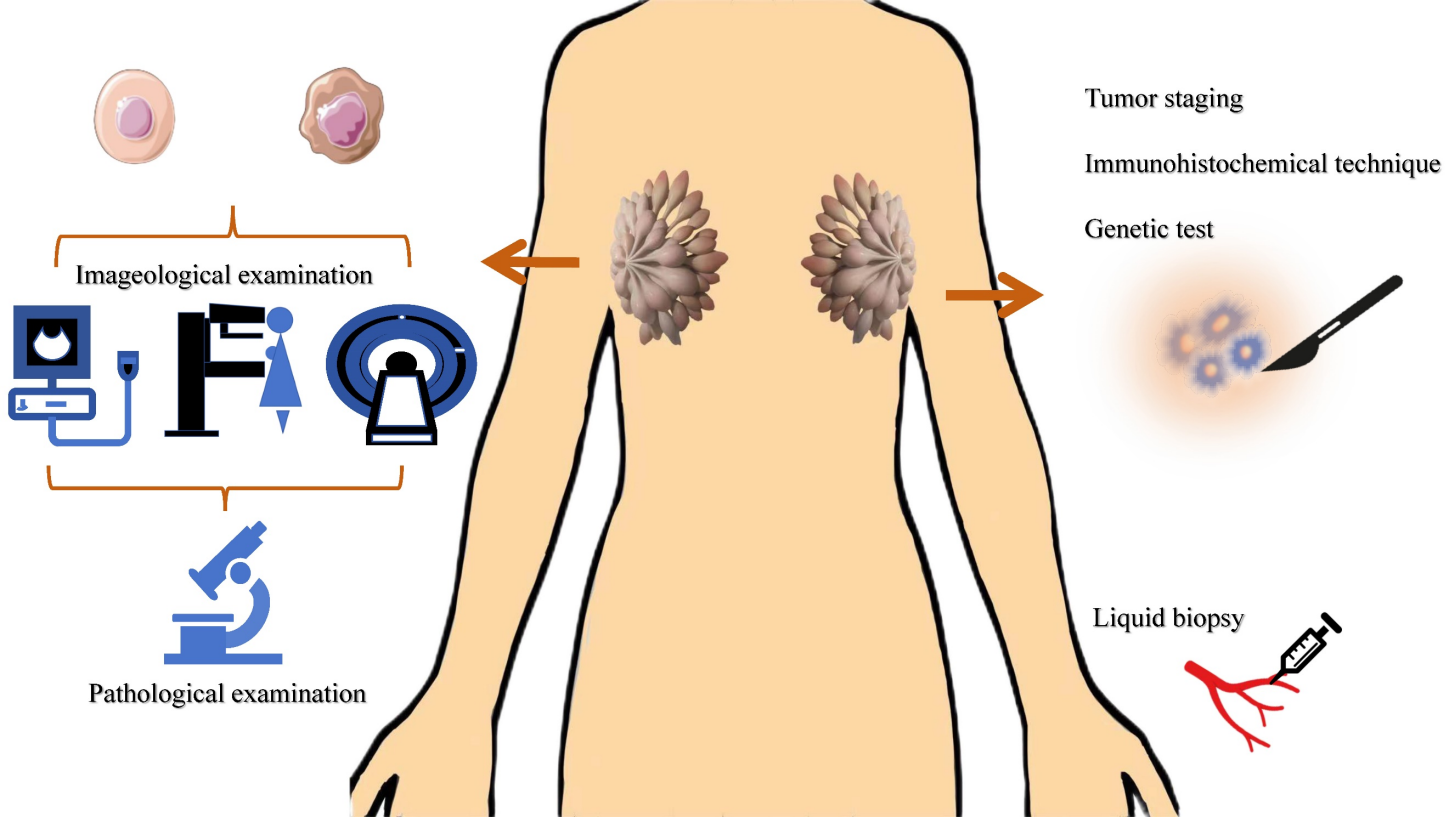
Common diagnostic methods of BC. The main methods for breast cancer screening are imaging tests. The molecular markers of breast cancer involve TNM staging, immunohistochemistry results, gene testing results, tumor marker detection, etc.

**Figure 3:**
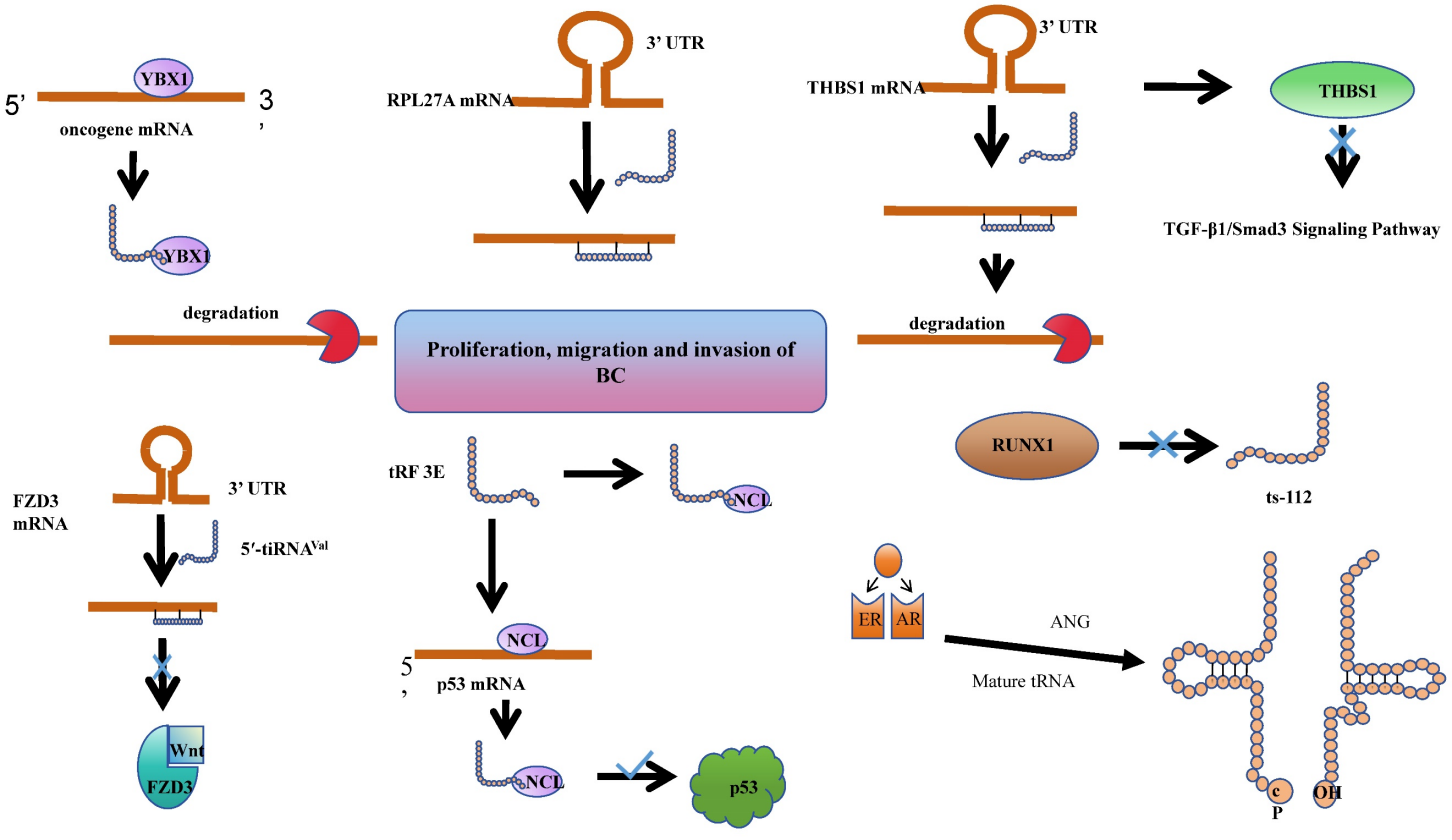
tsRNAs play roles in BC tumorigenesis and progression through various mechanisms.

**Figure 4:**
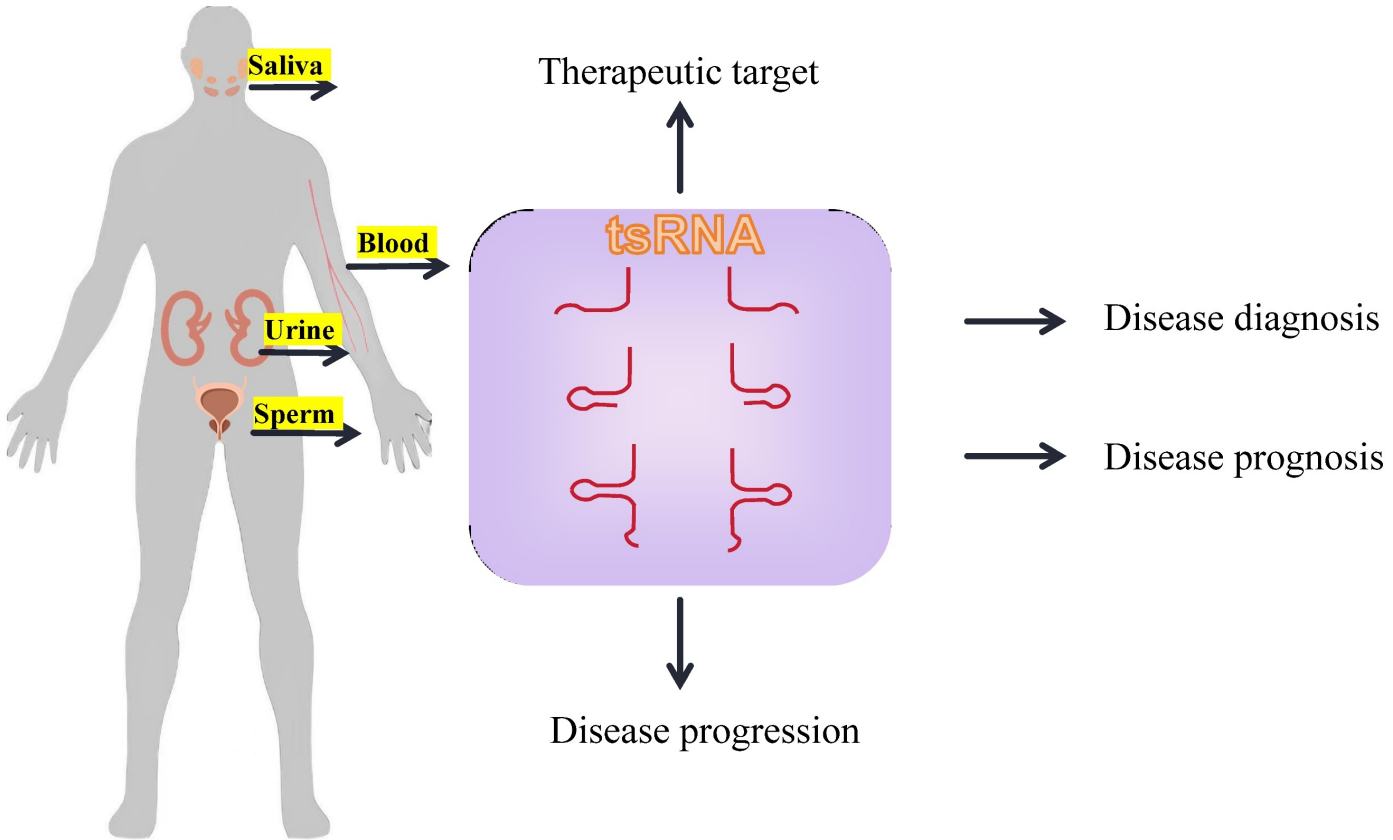
Common tsRNAs liquid biopsy in clinical applications.

**Table 1:** Clinical value of tsRNAs in BC

tsRNA	Symple	Expression level	Clinical signicance
tDR-7816^73^	Serum	Upregulated	Expression level has good diagnostic efficacy and was positively correlated with tumor size, lymph node metastasis, TNM stage and Ki-67 index
tRF-Gly-CCC-046, tRF-Tyr-GTA-010 and tRF-Pro-TGG-001^110^	Serum	Downregulated	Good diagnostic efficiency
tRF-30-JZOYJE22RR33 and tRF-27-ZDXPHO53KSN^111^	Serum	Upregulated	expression level was positively correlated with the resistance to trastuzumab
tRF-16-K8J7K1B^112^	Serum	Upregulated	Expression level was positively correlated with tamoxifen resistance and shorter disease-free survival in patients with hormone receptor positive (HR+) breast cancer
tDR-000620^76^	Serum	Downregulated	Expression level was negatively correlated with the RFS of breast cancer patients
tRF-Arg CCT 017 and tiRNA-Phe-GAA-003^74^	Serum	Upregulated	Expression level was positively correlated with DFS and OS in breast cancer patients
tRF2E^87^	Serum	Upregulated	Expression level was positively correlated with BC reflecting tumor status (control > early cancer > metastatic cancer)
tRF-19-W4PU732S^80^	Tissue	Upregulated	Expression level was positively correlated with OS in breast cancer patients
tRFdb-5024a, 5P_tRNA-Leu-CAA-4-1 and ts-49^113^	Tissue	Upregulated	Expression level was positively correlated with OS in breast cancer patients

## Abbreviations

ANG: Angiogenin
Apaf-1: Apoptotic protease activating factor 1
BC: Breast cancer
CDS: Coding sequence
CLP1: Cleavage factor polyribonucleotide kinase subunit 1
CSC: Cancer stem cells
Cyt c: Cytochrome c
DFS: Disease-free survival
EBC: Early breast cancer
EIF4F: Eukaryotic translation initiation factor 4F
EMT: Epithelial-to-mesenchymal transition
ERVs: Endogenous retroviruses
FAT4: FAT atypical cadherin 4
G6PC: Glucose 6-Phosphatase catalytic
HCC: Hepatocellular carcinoma
HGSOC: High-grade serous ovarian cancer
HSPB1: Heat shock protein family B member 1
HTLV-1: Human T-cell leukemia virus type 1
Igf2bp1: Insulin-like growth factor 2 mRNA-binding protein 1
JAG2: Jagged 2
LDHA: Lactate dehydrogenase A
LTR retrotransposons: Long terminal repeat retrotransposons
M1A: N1-methyladenosine
MED29: Mediator complex subunit 29
MERVL: Murine endogenous retrovirus-L
NCL: Nucleolin
NSCLC: Non-small cell lung cancer
OASL1: 2'-5'-oligoadenylate synthetase like Gene 1
OS: Overall survival
PABPC1: Polyadenylate-binding protein 1
PAR-Chip: Photoactivatable-ribonucleoside-enhanced crosslinking and immunoprecipitation
PBS: Primer binding site
PPM1F: Protein phosphatase, Mg2+/Mn2+ dependent 1F
PUS7: Pseudouridine synthase 7
RFS: Recurrence free survival
RISC: RNA-induced silencing complex
RNH1: Ribonuclease/angiogenin inhibitor 1
RSP28: Ribosomal protein 28
RSV: Respiratory syncytial virus
RUNX1: RUNX family transcription factor 1
SPEN: Split Ends
THBS1: Thrombospondin-1
TNBC: Triple-negative breast cancer
YBX1: Y-box binding protein 1

## References

[1] Fu M, J Gu, M Wang (2023). Emerging roles of tRNA-derived fragments in cancer. Mol Cancer.

[2] Zhang Y, L Ren, X Sun (2021). Angiogenin mediates paternal inflammation-induced metabolic disorders in offspring through sperm tsRNAs. Nat Commun.

[3] Londin E, R Magee, CL Shields (2020). IsomiRs and tRNA-derived fragments are associated with metastasis and patient survival in uveal melanoma. Pigment Cell Melanoma Res.

[4] Veneziano D, L Tomasello, V Balatti (2019). Dysregulation of different classes of tRNA fragments in chronic lymphocytic leukemia. Proc Natl Acad Sci U S A.

[5] Chen Q, X Zhang, J Shi (2021). Origins and evolving functionalities of tRNA-derived small RNAs. Trends Biochem Sci.

[6] Fagan SG, M Helm, JHM Prehn (2021). tRNA-derived fragments: A new class of non-coding RNA with key roles in nervous system function and dysfunction. Prog Neurobiol.

[7] Li YK, LR Yan, A Wang (2022). RNA-sequencing reveals the expression profiles of tsRNAs and their potential carcinogenic role in cholangiocarcinoma. J Clin Lab Anal.

[8] Xu C, Y Fu (2021). Expression Profiles of tRNA-Derived Fragments and Their Potential Roles in Multiple Myeloma. Onco Targets Ther.

[9] Zhang L, J Liu, Y Hou (2023). Classification, function, and advances in tsRNA in non-neoplastic diseases. Cell Death Dis.

[10] Barzaman K, J Karami, Z Zarei (2020). Breast cancer: Biology, biomarkers, and treatments. Int Immunopharmacol.

[11] Bevers TB, M Helvie, E Bonaccio (2018). Breast Cancer Screening and Diagnosis, Version 3.2018, NCCN Clinical Practice Guidelines in Oncology. J Natl Compr Canc Netw.

[12] Torre LA, RL Siegel, EM Ward (2016). Global Cancer Incidence and Mortality Rates and Trends—An Update. Cancer Epidemiology, Biomarkers & Prevention.

[13] Sung H, J Ferlay, RL Siegel (2021). Global Cancer Statistics 2020: GLOBOCAN Estimates of Incidence and Mortality Worldwide for 36 Cancers in 185 Countries. CA Cancer J Clin.

[14] Phizicky EM, AK Hopper (2010). tRNA biology charges to the front. Genes Dev.

[15] Maraia RJ, TN Lamichhane (2010). 3′ processing of eukaryotic precursor tRNAs. WIREs RNA.

[16] Aravin AA, M Lagos-Quintana, A Yalcin (2003). The small RNA profile during Drosophila melanogaster development. Dev Cell.

[17] Jochl C, M Rederstorff, J Hertel (2008). Small ncRNA transcriptome analysis from Aspergillus fumigatus suggests a novel mechanism for regulation of protein synthesis. Nucleic Acids Res.

[18] Diallo I, J Ho, M Lambert (2022). A tRNA-derived fragment present in E. coli OMVs regulates host cell gene expression and proliferation. PLoS Pathog.

[19] Liu B, J Cao, X Wang (2021). Deciphering the tRNA-derived small RNAs: origin, development, and future. Cell Death Dis.

[20] Di Fazio A, M Gullerova (2023). An old friend with a new face: tRNA-derived small RNAs with big regulatory potential in cancer biology. Br J Cancer.

[21] Sobala A, G Hutvagner (2011). Transfer RNA-derived fragments: origins, processing, and functions. Wiley Interdiscip Rev RNA.

[22] Lee YS, Y Shibata, A Malhotra (2009). A novel class of small RNAs: tRNA-derived RNA fragments (tRFs). Genes & Development.

[23] Kumar P, C Kuscu, A Dutta (2016). Biogenesis and Function of Transfer RNA-Related Fragments (tRFs). Trends in Biochemical Sciences.

[24] Kumar P, SB Mudunuri, J Anaya (2015). tRFdb: a database for transfer RNA fragments. Nucleic Acids Res.

[25] Telonis AG, P Loher, S Honda (2015). Dissecting tRNA-derived fragment complexities using personalized transcriptomes reveals novel fragment classes and unexpected dependencies. Oncotarget.

[26] Babiarz JE, JG Ruby, Y Wang (2008). Mouse ES cells express endogenous shRNAs, siRNAs, and other Microprocessor-independent, Dicer-dependent small RNAs. Genes Dev.

[27] Kumar P, J Anaya, SB Mudunuri (2014). Meta-analysis of tRNA derived RNA fragments reveals that they are evolutionarily conserved and associate with AGO proteins to recognize specific RNA targets. BMC Biol.

[28] Huang B, H Yang, X Cheng (2017). tRF/miR-1280 Suppresses Stem Cell-like Cells and Metastasis in Colorectal Cancer. Cancer Res.

[29] Lee SR, K Collins (2005). Starvation-induced cleavage of the tRNA anticodon loop in Tetrahymena thermophila. J Biol Chem.

[30] Kim HK, JH Yeom, MA Kay (2020). Transfer RNA-Derived Small RNAs: Another Layer of Gene Regulation and Novel Targets for Disease Therapeutics. Mol Ther.

[31] Saikia M, D Krokowski, BJ Guan (2012). Genome-wide identification and quantitative analysis of cleaved tRNA fragments induced by cellular stress. J Biol Chem.

[32] Krishna S, DG Yim, V Lakshmanan (2019). Dynamic expression of tRNA-derived small RNAs define cellular states. EMBO Rep.

[33] Mishima E, C Inoue, D Saigusa (2014). Conformational change in transfer RNA is an early indicator of acute cellular damage. J Am Soc Nephrol.

[34] Tao EW, HL Wang, WY Cheng (2021). A specific tRNA half, 5'tiRNA-His-GTG, responds to hypoxia via the HIF1alpha/ANG axis and promotes colorectal cancer progression by regulating LATS2. J Exp Clin Cancer Res.

[35] Cui Y, Y Huang, X Wu (2019). Hypoxia-induced tRNA-derived fragments, novel regulatory factor for doxorubicin resistance in triple-negative breast cancer. J Cell Physiol.

[36] Elkordy A, E Mishima, K Niizuma (2018). Stress-induced tRNA cleavage and tiRNA generation in rat neuronal PC12 cells. J Neurochem.

[37] Rashad S, T Tominaga, K Niizuma (2021). The cell and stress-specific canonical and noncanonical tRNA cleavage. J Cell Physiol.

[38] Honda S, P Loher, M Shigematsu (2015). Sex hormone-dependent tRNA halves enhance cell proliferation in breast and prostate cancers. Proc Natl Acad Sci U S A.

[39] Wang JH, WX Chen, SQ Mei (2022). tsRFun: a comprehensive platform for decoding human tsRNA expression, functions and prognostic value by high-throughput small RNA-Seq and CLIP-Seq data. Nucleic Acids Res.

[40] Vigneault F, D Ter-Ovanesyan, S Alon (2012). High-throughput multiplex sequencing of miRNA. Curr Protoc Hum Genet.

[41] Kumar P, C Kuscu, A Dutta (2016). Biogenesis and Function of Transfer RNA-Related Fragments (tRFs). Trends Biochem Sci.

[42] Li S, Z Xu, J Sheng (2018). tRNA-Derived Small RNA: A Novel Regulatory Small Non-Coding RNA. Genes (Basel).

[43] Wu J, H Xu, F Hu (2023). CRISPR-Cas and catalytic hairpin assembly technology for target-initiated amplification detection of pancreatic cancer specific tsRNAs. Front Bioeng Biotechnol.

[44] Xie Y, L Yao, X Yu (2020). Action mechanisms and research methods of tRNA-derived small RNAs. Signal Transduct Target Ther.

[45] Zhang Y, X Gu, Y Li (2024). Multiple regulatory roles of the transfer RNA-derived small RNAs in cancers. Genes Dis.

[46] Gan L, H Song, X Ding (2023). Transfer RNA-derived small RNAs (tsRNAs) in gastric cancer. Front Oncol.

[47] Gagnon Keith T, L Li, Y Chu (2014). RNAi Factors Are Present and Active in Human Cell Nuclei. Cell Reports.

[48] Pekarsky Y, V Balatti, A Palamarchuk (2016). Dysregulation of a family of short noncoding RNAs, tsRNAs, in human cancer. Proc Natl Acad Sci U S A.

[49] Zhang X, X He, C Liu (2016). IL-4 Inhibits the Biogenesis of an Epigenetically Suppressive PIWI-Interacting RNA To Upregulate CD1a Molecules on Monocytes/Dendritic Cells. J Immunol.

[50] Maute RL, C Schneider, P Sumazin (2013). tRNA-derived microRNA modulates proliferation and the DNA damage response and is down-regulated in B cell lymphoma. Proc Natl Acad Sci U S A.

[51] Zhang M, F Li, J Wang (2019). tRNA-derived fragment tRF-03357 promotes cell proliferation, migration and invasion in high-grade serous ovarian cancer. Onco Targets Ther.

[52] Shen L, Z Tan, M Gan (2019). tRNA-Derived Small Non-Coding RNAs as Novel Epigenetic Molecules Regulating Adipogenesis. Biomolecules.

[53] Luan N, Y Chen, Q Li (2021). TRF-20-M0NK5Y93 suppresses the metastasis of colon cancer cells by impairing the epithelial-to-mesenchymal transition through targeting Claudin-1. Am J Transl Res.

[54] Sharma U, CC Conine, JM Shea (2016). Biogenesis and function of tRNA fragments during sperm maturation and fertilization in mammals. Science.

[55] Yang W, K Gao, Y Qian (2022). A novel tRNA-derived fragment AS-tDR-007333 promotes the malignancy of NSCLC via the HSPB1/MED29 and ELK4/MED29 axes. J Hematol Oncol.

[56] Kim HK, G Fuchs, S Wang (2017). A transfer-RNA-derived small RNA regulates ribosome biogenesis. Nature.

[57] Keam SP, A Sobala, S Ten Have (2017). tRNA-Derived RNA Fragments Associate with Human Multisynthetase Complex (MSC) and Modulate Ribosomal Protein Translation. J Proteome Res.

[58] Guzzi N, M Ciesla, PCT Ngoc (2018). Pseudouridylation of tRNA-Derived Fragments Steers Translational Control in Stem Cells. Cell.

[59] Blanco S, S Dietmann, JV Flores (2014). Aberrant methylation of tRNAs links cellular stress to neuro-developmental disorders. EMBO J.

[60] Ivanov P, MM Emara, J Villen (2011). Angiogenin-induced tRNA fragments inhibit translation initiation. Mol Cell.

[61] Ivanov P, E O'Day, MM Emara (2014). G-quadruplex structures contribute to the neuroprotective effects of angiogenin-induced tRNA fragments. Proceedings of the National Academy of Sciences.

[62] Tao EW, WY Cheng, WL Li (2020). tiRNAs: A novel class of small noncoding RNAs that helps cells respond to stressors and plays roles in cancer progression. J Cell Physiol.

[63] Franchini G, C Nicot, JM Johnson (2003). Seizing of T cells by human T-cell leukemia/lymphoma virus type 1. Adv Cancer Res.

[64] Ruggero K, A Guffanti, A Corradin (2014). Small noncoding RNAs in cells transformed by human T-cell leukemia virus type 1: a role for a tRNA fragment as a primer for reverse transcriptase. J Virol.

[65] Wilhelm M, FX Wilhelm (2001). Reverse transcription of retroviruses and LTR retrotransposons. Cell Mol Life Sci.

[66] Schorn AJ, MJ Gutbrod, C LeBlanc (2017). LTR-Retrotransposon Control by tRNA-Derived Small RNAs. Cell.

[67] Shao Y, Q Sun, X Liu (2017). tRF-Leu-CAG promotes cell proliferation and cell cycle in non-small cell lung cancer. Chem Biol Drug Des.

[68] Saikia M, R Jobava, M Parisien (2014). Angiogenin-cleaved tRNA halves interact with cytochrome c, protecting cells from apoptosis during osmotic stress. Mol Cell Biol.

[69] Li K, Y Lin, Y Luo (2022). A signature of saliva-derived exosomal small RNAs as predicting biomarker for esophageal carcinoma: a multicenter prospective study. Mol Cancer.

[70] Selitsky SR, J Baran-Gale, M Honda (2015). Small tRNA-derived RNAs are increased and more abundant than microRNAs in chronic hepatitis B and C. Sci Rep.

[71] Gu X, S Ma, B Liang (2021). Serum hsa_tsr016141 as a Kind of tRNA-Derived Fragments Is a Novel Biomarker in Gastric Cancer. Front Oncol.

[72] Goodarzi H, X Liu, Hoang CB Nguyen (2015). Endogenous tRNA-Derived Fragments Suppress Breast Cancer Progression via YBX1 Displacement. Cell.

[73] Huang Y, H Ge, M Zheng (2020). Serum tRNA-derived fragments (tRFs) as potential candidates for diagnosis of nontriple negative breast cancer. J Cell Physiol.

[74] Wang J, G Ma, H Ge (2021). Circulating tRNA-derived small RNAs (tsRNAs) signature for the diagnosis and prognosis of breast cancer. NPJ Breast Cancer.

[75] Wang J, G Ma, M Li (2020). Plasma tRNA Fragments Derived from 5' Ends as Novel Diagnostic Biomarkers for Early-Stage Breast Cancer. Mol Ther Nucleic Acids.

[76] Feng W, Y Li, J Chu (2018). Identification of tRNA-derived small noncoding RNAs as potential biomarkers for prediction of recurrence in triple-negative breast cancer. Cancer Med.

[77] Zhu P, J Lu, X Zhi (2021). tRNA-derived fragment tRFLys-CTT-010 promotes triple-negative breast cancer progression by regulating glucose metabolism via G6PC. Carcinogenesis.

[78] Zhang YN, KR Xia, CY Li (2021). Review of Breast Cancer Pathologigcal Image Processing. Biomed Res Int.

[79] Yager JD, NE Davidson (2006). Estrogen carcinogenesis in breast cancer. N Engl J Med.

[80] Zhang Z, Z Liu, W Zhao (2022). tRF-19-W4PU732S promotes breast cancer cell malignant activity by targeting inhibition of RPL27A (ribosomal protein-L27A). Bioengineered.

[81] Liu X, W Mei, V Padmanaban (2022). A pro-metastatic tRNA fragment drives Nucleolin oligomerization and stabilization of its bound metabolic mRNAs. Mol Cell.

[82] Chen F, C Song, F Meng (2023). 5'-tRF-GlyGCC promotes breast cancer metastasis by increasing fat mass and obesity-associated protein demethylase activity. Int J Biol Macromol.

[83] Mo D, P Jiang, Y Yang (2019). A tRNA fragment, 5'-tiRNA(Val), suppresses the Wnt/beta-catenin signaling pathway by targeting FZD3 in breast cancer. Cancer Lett.

[84] Mo D, F He, J Zheng (2021). tRNA-Derived Fragment tRF-17-79MP9PP Attenuates Cell Invasion and Migration via THBS1/TGF-beta1/Smad3 Axis in Breast Cancer. Front Oncol.

[85] Wang B, D Li, Y Ilnytskyy (2022). A miR-34a-guided, tRNA(i)(Met)-derived, piR_019752-like fragment (tRiMetF31) suppresses migration and angiogenesis of breast cancer cells via targeting PFKFB3. Cell Death Discov.

[86] Asif HM, S Sultana, S Ahmed (2016). HER-2 Positive Breast Cancer - a Mini-Review. Asian Pac J Cancer Prev.

[87] Falconi M, M Giangrossi, ME Zabaleta (2019). A novel 3'-tRNA(Glu)-derived fragment acts as a tumor suppressor in breast cancer by targeting nucleolin. FASEB J.

[88] Akram M, M Iqbal, M Daniyal (2017). Awareness and current knowledge of breast cancer. Biol Res.

[89] Farina NH, S Scalia, CE Adams (2020). Identification of tRNA-derived small RNA (tsRNA) responsive to the tumor suppressor, RUNX1, in breast cancer. J Cell Physiol.

[90] Gadaleta E, GJ Thorn, H Ross-Adams (2022). Field cancerization in breast cancer. J Pathol.

[91] He Z, Z Chen, M Tan (2020). A review on methods for diagnosis of breast cancer cells and tissues. Cell Prolif.

[92] Pruthi S, KR Brandt, AC Degnim (2007). A multidisciplinary approach to the management of breast cancer, part 1: prevention and diagnosis. Mayo Clin Proc.

[93] Libson S, M Lippman (2014). A review of clinical aspects of breast cancer. Int Rev Psychiatry.

[94] Donepudi MS, K Kondapalli, SJ Amos (2014). Breast cancer statistics and markers. J Cancer Res Ther.

[95] Rakha EA, AR Green (2017). Molecular classification of breast cancer: what the pathologist needs to know. Pathology.

[96] He C, S Zheng, Y Luo (2018). Exosome Theranostics: Biology and Translational Medicine. Theranostics.

[97] Weng Q, Y Wang, Y Xie (2022). Extracellular vesicles-associated tRNA-derived fragments (tRFs): biogenesis, biological functions, and their role as potential biomarkers in human diseases. J Mol Med (Berl).

[98] Chiou NT, R Kageyama, KM Ansel (2018). Selective Export into Extracellular Vesicles and Function of tRNA Fragments during T Cell Activation. Cell Rep.

[99] Gambaro F, M Li Calzi, P Fagundez (2020). Stable tRNA halves can be sorted into extracellular vesicles and delivered to recipient cells in a concentration-dependent manner. RNA Biol.

[100] Zhu L, J Li, Y Gong (2019). Exosomal tRNA-derived small RNA as a promising biomarker for cancer diagnosis. Mol Cancer.

[101] Lu S, X Wei, L Tao (2022). A novel tRNA-derived fragment tRF-3022b modulates cell apoptosis and M2 macrophage polarization via binding to cytokines in colorectal cancer. J Hematol Oncol.

[102] Schageman J, E Zeringer, M Li (2013). The complete exosome workflow solution: from isolation to characterization of RNA cargo. Biomed Res Int.

[103] Yuan T, X Huang, M Woodcock (2016). Plasma extracellular RNA profiles in healthy and cancer patients. Sci Rep.

[104] Dhahbi JM SR Spindler, H Atamna (2013). 5'-YRNA fragments derived by processing of transcripts from specific YRNA genes and pseudogenes are abundant in human serum and plasma. Physiol Genomics.

[105] Srinivasan S, A Yeri, PS Cheah (2019). Small RNA Sequencing across Diverse Biofluids Identifies Optimal Methods for exRNA Isolation. Cell.

[106] Akat KM, YA Lee, A Hurley (2019). Detection of circulating extracellular mRNAs by modified small-RNA-sequencing analysis. JCI Insight.

[107] Dhahbi JM SR Spindler, H Atamna (2013). 5' tRNA halves are present as abundant complexes in serum, concentrated in blood cells, and modulated by aging and calorie restriction. BMC Genomics.

[108] Olvedy M, M Scaravilli, Y Hoogstrate (2016). A comprehensive repertoire of tRNA-derived fragments in prostate cancer. Oncotarget.

[109] Wang J, X Liu, W Cui (2022). Plasma tRNA-derived small RNAs signature as a predictive and prognostic biomarker in lung adenocarcinoma. Cancer Cell Int.

[110] Zhang Y, Z Bi, X Dong (2021). tRNA-derived fragments: tRF-Gly-CCC-046, tRF-Tyr-GTA-010 and tRF-Pro-TGG-001 as novel diagnostic biomarkers for breast cancer. Thorac Cancer.

[111] Sun C, F Yang, Y Zhang (2018). tRNA-Derived Fragments as Novel Predictive Biomarkers for Trastuzumab-Resistant Breast Cancer. Cell Physiol Biochem.

[112] Sun C, X Huang, J Li (2023). Exosome-Transmitted tRF-16-K8J7K1B Promotes Tamoxifen Resistance by Reducing Drug-Induced Cell Apoptosis in Breast Cancer. Cancers (Basel).

[113] Shan N, N Li, Q Dai (2020). Interplay of tRNA-Derived Fragments and T Cell Activation in Breast Cancer Patient Survival. Cancers (Basel).

